# Dementia and associated factors among the elderly in Vietnam: a cross-sectional study

**DOI:** 10.1186/s13033-019-0314-7

**Published:** 2019-08-23

**Authors:** Nguyen Ngoc Bich, Nguyen Thi Thuy Dung, Tran Vu, Lam Thi Quy, Nguyen Anh Tuan, Nguyen Thi Thanh Binh, Nguyen Trong Hung, Le Vu Anh

**Affiliations:** 1 Hanoi University of Public Health, Hanoi, Vietnam; 2 Vietnam Public Health Association, Hanoi, Vietnam; 3 Hanoi Medical University, Hanoi, Vietnam; 4 National Gerontology Hospital, Hanoi, Vietnam

**Keywords:** Dementia, Cognitive symptoms, Elderly, Vietnam, MMSE, Associated factors, Prevalence

## Abstract

**Background:**

Dementia poses a serious threat to the wellbeing of the elderly. In the context of the rapidly ageing population of Vietnam however, little is known about the prevalence of symptoms and other related factors. This study aims to detect the prevalence of cognitive symptoms of dementia in the elderly in Vietnam as well as other associated factors.

**Methods:**

A cross-sectional study was conducted over a period of six communes at the Northern, Central and Southern region of Vietnam. Prevalence of cognitive symptoms of dementia was the outcome of interest and assessed by Mini Mental State Evaluation (MMSE) questionnaire and was standardized according to the age structure of Vietnam. A total of 3308 adults aged 60 and above were included. Association between having cognitive symptoms of dementia and other factors was assessed with logistic regression.

**Findings:**

Cognitive symptoms of dementia were perceived in 46.4% of the sample group. The symptoms were more common among participants who were older, female, had a lower educational level, were not physically active or have previously had stroke.

**Conclusions:**

Prevalence of cognitive symptoms of dementia in adults aged 60 and above was relatively high in Vietnam. Other modifiable associated factors including physical inactivity and social connectedness should also be considered in designing intervention program to prevent dementia in the future.

## Background

Dementia is a syndrome of deterioration in memory, thoughts, behaviours and the ability to perform daily activities. Previous studies showed that older adults suffering from dementia, had to face social stigma due to lack of awareness of community members about the syndrome [1]. Dementia is prevalent worldwide with 44.35 million patients in 2013 and projected 75.62 million patients in 2030 [1]. There is a wide range of risk factors associated with dementia such as smoking, alcohol abuse, and other non-communicable diseases. The risk of having dementia increased in older groups especially from the age of 70 or above [2, 3]. National survey conducted by General Statistic Office showed that Vietnam population became “Aging population” as older people occupied more than 10% of the total population. According to Vietnam Aging Survey, Vietnam’s older population reported higher prevalence of non-communicable diseases. Smoking and alcohol abuse was also more common among older males [4]. In Vietnam, dementia or mental health in general has not been recognised as a health priority. There are not many studies available that have estimated the magnitude of dementia occurrence in Vietnam and especially there has not been a large-scale population study to capture the prevalence of the syndrome among high-risk groups, i.e., individuals older than 60 years old. There were some primary studies conducted in Vietnam with a small sample. These studies estimated that the prevalence of dementia was around 4.5% [2, 5–7]. There is, however, a dearth of statistics of associated factors with dementia in a Vietnamese context. Different subtypes of dementia are characterised with different sets of symptoms but cognitive impairment is the most early sign of the syndrome [8]. It has been suggested that cognitive impairment is an intermediate stage between normal cognition and dementia [9]. This study aims to investigate the prevalence of cognitive symptoms of dementia and its associated factors in adults aged 60 and above in Vietnam.

## Method

### Design and setting

This cross-sectional study was conducted in six communes in six provinces of Vietnam in 2016. A commune is the third administrative tier of Vietnam’s administrative hierarchy, after the province and district levels. The six communes were conveniently selected to cover urban and rural areas of the Northern, Central and Southern Vietnam.

### Participants and sample size

Participants of the study were elderly people living in the study sites and met our inclusion criteria of (1) being 60 years old or above; (2) being a registered resident in the designated communes and (3) not living with any diagnosed mental disorder that could compromise the autonomy to participate in the study.

Before the recruitment of participants, each commune health centre conducted a review in the population management records of the commune to create a sampling frame which included 5539 adults over the age of 60. Of those, 1340 were absent or moved without registering with the authority. Total of 4199 individuals were reached and invited to participate in the study. Among those, 891 refused to participate. No information about people who refused or were absent is available. A total of 3308 respondents gave consent to have their information collected and to be interviewed.

### Data collection

Data were obtained using a structured questionnaire. Interviews were conducted by trained interviewers who were local health staff and were not involved in any other aspects of the study, such as recruiting participants or analysing data. All interviewers participated in a half-day training by a member of the research group on using the questionnaire and the importance of ethics in human research such as confidentiality of the data collected. Each interview was conducted in a private room with an interviewer and the participant. Where the participants needed assistance from another family member to answer demographic questions, another member of the family or their caregiver was allowed to be present in the room. Each participant received 30.000 Vietnam Dong (VND) ($1.3) as compensation for the time attending the study.

### Variables and measurements

Cognitive symptoms of dementia were assessed by the MMSE questionnaire—in Vietnamese. This questionnaire was used globally as the tool to measure cognitive functions and has high accuracy in detecting dementia. The MMSE includes 11 questions to assess 7 aspects including Orientation, Registration, Attention and Calculation, Recall, Language, Repetition and Complex commands, however, for the purpose of this study only the total score is examined. The total score ranges from 0 to 30. The higher the score, the better the cognitive function of the participant. Participants who scored less than 24 were categorized as having dementia cognitive symptoms in this study. This cut-off point was widely used in the studies in the scientific community and was suggested as having optimal sensitivity (0.85) and specificity (0.90) [10].

### Associated factors

According to previous studies, ten potential associated factors with dementia, self-reported by participants or by caregivers, were included in the analyses:

1. Sex: male and female.
2. Age: categorised into five groups of 60 to 64, 65 to 69, 70 to 74, 75 to 79 and 80 years or above.
3. Highest level of education: categorized into five groups according to the Vietnamese educational system consisting of illiteracy, primary school, secondary school, high school or vocational school, and higher than high school (i.e., college or higher).
4. Main caregiver: was defined as the main person who cares for the participants, living either in the same accommodation or nearby.
5. Diet: was assessed by asking if the participant’s current diet consisted of mixed elements (i.e., mixed meat, vegetables, grains), mainly vegetable, mainly seafood or following a weightless diet.
6. Physical activity level: was assessed by asking participants if they were currently engaging in any habitual or occupational physical activity at any intensity level of including daily exercise or physical activity at work.
7. History of high blood pressure: self-reported.
8. History of stroke: self-reported.
9. Frequency participating in social activities per week or month: self-reported.
10. Frequency visiting friends or neighbours per week or month: self-reported.

### Statistical method

Characteristics of participants were described as a total sample and stratified by status, number and proportion of cognitive symptoms of dementia. Logistic regression was used to assess the factors associated with cognitive symptoms of dementia. Two types of model were developed: (1) crude model included one factor of interest and the outcome; and (2) adjusted model that included all potential associated factors. Odds ratios and its 95% confidence interval were reported. Data was entered using Epi Data 3.1 and analysed using SPSS 16.0.

Ethical consideration of the study was reviewed and approved by the ethics committee of Hanoi University of Public Health (decision number 240/2015/YTCC-HD in 2015). Participants were provided with information about the study including purpose, procedure and risks. Written consent forms were signed and given by all participants. For participants who had difficulties in communication or were not able to read the form, their caregivers signed the consent form on their behalf and assisted them during the interview.

## Results

### Participant characteristics and prevalence of dementia cognitive symptoms

Table 1 shows characteristics of the participant as total and stratified by cognitive symptoms of dementia. Of the total 3308 participants (age ranged from 60 to 103, mean = 71.9), majority were between 60 and 70 years old (47%), about 60% were female and had primary schools as their education attainment (53%), the illiterate proportion was 8.8%. More than 50% of participants were non-religious and about one fourth were practicing Buddhist. Majority were Kinh people, the dominant ethnicity in Vietnam, only 2.1% were minorities (data not shown). The main occupations were agriculture, forestry or fishery, accounting for 55% (data not shown).

**Table 1:** Participant characteristics

Characteristics (n = 3308)	Total sample, n (%^a^)	Dementia symptoms, n (%^b^)	
		No (n = 1717)	Yes (n = 1591)
Sex*			
Male	1333 (40.3)	846 (63.5)	487 (36.5)
Female	1975 (59.7)	871 (44.1)	1104 (55.9)
Age group*			
60–64	802 (24.2)	597 (74.4)	205 (25.6)
65–69	738 (22.3)	476 (64.5)	262 (35.5)
70–74	566 (17.1)	304 (53.7)	262 (46.3)
75–79	516 (15.6)	215 (41.7)	301 (58.3)
80+	681 (20.6)	121 (17.8)	560 (82.2)
Education*			
Illiteracy	290 (8.8)	15 (5.2)	275 (94.8)
Primary school	1754 (53.0)	712 (40.6)	1024 (59.4)
Secondary school	591 (17.9)	413 (72.9)	160 (27.1)
High school/vocational	463 (14.0)	376 (81.2)	87 (18.8)
Higher than high school	210 (6.3)	183 (87.1)	27 (12.9)
Main caregiver*			
Self-care	1838 (55.6)	1013 (55.1)	825 (44.9)
Spouse	701 (21.2)	448 (63.9)	253 (36.1)
Children	690 (20.9)	230 (33.3)	460 (66.7)
Others (neighbour, cousin…)	79 (2.4)	26 (32.9)	53 (67.1)
Having mixed diet*			
No	1190 (36.0)	525 (44.1)	665 (55.9)
Yes	2118 (64.0)	1192 (69.4)	926 (58.2)
Physically active*			
No	532 (16.1)	132 (51.9)	400 (75.2)
Yes	2776 (83.9)	1585 (92.3)	1191 (74.9)
Participating in social activities*			
Daily	154 (4.7)	122 (79.2)	32 (20.8)
3–4 times/week	45 (1.4)	35 (77.8)	10 (22.2)
1–2 times/week	62 (1.9)	43 (69.4)	19 (30.6)
1–2 times/month	658 (19.9)	385 (58.5)	273 (41.5)
Less than 1 time/month	552 (16.7)	318 (57.6)	234 (42.4)
Never	1837 (55.5)	814 (44.3)	1023 (55.7)
Visiting friends, neighbours*			
Daily	1249 (37.8)	752 (60.2)	497 (39.8)
3–4 times/week	401 (12.1)	213 (53.1)	188 (46.9)
1–2 times/week	503 (15.2)	269 (53.5)	234 (46.5)
1–2 times/month	327 (9.9)	186 (56.9)	141 (43.1)
Less than 1 time/month	271 (8.2)	130 (48.0)	141 (52.0)
Never	557 (16.8)	167 (30.0)	390 (70.0)
High blood pressure*			
No	2055 (62.1)	1104 (53.7)	951 (46.3)
Yes	1253 (37.9)	613 (35.7)	640 (40.2)
Stroke*			
No	3159 (95.5)	1678 (53.1)	1481 (46.9)
Yes	149 (4.5)	39 (2.3)	110 (6.9)

* Significantly different between groups with and without symptoms of dementia (χ^2^ p-value < 0.05)

^a^Percentage by column

^b^Percentage by row

Using the MMSE questionnaire to detect dementia among elderly participants, the average score was 22.3. There were 59 participants (1.8%) who got a score of 0 and 175 participants (5.3%) got the maximum point of 30. Using the cut-off point of 24, 48% of participants were categorized as having cognitive symptoms of dementia. After standardizing the sample following the age structure of Vietnam in 2013 [11], the figure was slightly reduced to 46.4%.

Age and sex are significantly correlated with cognitive symptoms of dementia. The older the age group, the higher the prevalence of the symptoms. The age group of 60 to 64 had about 25% with dementia while this percentage among the group 80+ was more than three folds (82%). In this study, female participants had two times higher odds of having cognitive symptoms of dementia than male participants. There was a great difference in the proportions of participants with symptoms where level of education was assessed, with 95% symptoms observed in the lowest education groups as well as illiterate individuals, compared with 13% in the ones with higher levels of education. Those who were cared for themselves or were taken care of by their spouses, had a lower percentage of having cognitive symptoms of dementia compared to those who were looked after by their children and others (less than 45% compared to more than 65%).

Being physically inactive is also associated with having cognitive symptoms of dementia. The proportion of participants with symptoms was 1.8 time higher in physically inactive groups than in the active groups. Participating in social activities like community club, fund raising and visiting friends and neighbours also associates with lower prevalence of cognitive symptoms. Proportion of having the symptoms was 56% among the group who never took part in any activity while the figure in the group who did on a daily basis was only 21%. With a similar trend, 40% of the participants who visited friends and neighbours every day, had cognitive symptoms of dementia but this number among those who visited was at 70%. The symptoms were more common among groups who had high blood pressure or stroke.

### Associated factors of dementia

Table 2 shows the results from logistic regressions for cognitive symptoms of dementia. Bivariate analysis indicated that age, sex, educational level, main caregiver, mixed diet, level of physical activity, engaging in social activities, frequency of engaging in social activities, blood pressure level and history of stroke were associated with higher cognitive symptoms.

**Table 2:** Factors associated with dementia symptoms

Characteristics	Crude models		Adjusted model	
	OR	95% CI	OR	95% CI
Age group				
60–64	1	–	1	–
65–69	1.60^a^	1.29–2.00	1.60	1.24–2.06
70–74	2.51^a^	2.00–3.16	2.77^a^	2.13–3.62
75–79	4.08^a^	3.22–5.16	3.81^a^	2.89–5.03
80+	13.48^a^	10.47–17.35	9.42^a^	6.98–12.71
Sex				
Male	1	–	1	–
Female	2.20^a^	1.91–2.54	1.70^a^	1.41–2.04
Education				
Illiteracy	1	–	1	–
Primary school	0.08^a^	0.05–0.13	0.09^a^	0.05–0.15
Secondary school	0.02^a^	0.01–0.03	0.03^a^	0.02–0.05
High school/vocational	0.01^a^	0.01–0.02	0.02^a^	0.01–0.03
Higher than high school	0.01^a^	0.004–0.02	0.01^a^	0.006–0.03
Main caregiver				
Self-care	1	–	1	–
Spouse	0.69^a^	0.58–0.83	1.36^b^	1.07–1.72
Children	2.46^a^	2.04–2.95	1.35^b^	1.07–1.72
Others (neighbours, cousin…)	2.50^a^	1.55–4.04	0.97	0.52–1.78
Mixed diet				
No	1	–	1	–
Yes	0.61^a^	0.53–0.71	0.88	0.73–1.05
Physically active				
Yes	1	–	1	–
No	4.03^a^	3.27–4.98	1.89^a^	1.45–2.49
Participating social activities				
Daily	1	–	1	–
3–4 times/week	1.09	0.49–2.43	1.30	0.51–3.31
1–2 times/week	1.68	0.87–3.28	1.86	0.85–4.09
1–2 times/month	2.70^a^	1.78–4.11	2.08^b^	1.28–3.39
Less than 1 time/month	2.80^a^	1.83–4.29	1.36	0.83–2.25
Never	4.79^a^	3.21–7.15	2.08^b^	1.30–3.34
Visiting friends, neighbours				
Daily	1	–	1	–
3–4 times/week	1.33^b^	1.06–1.67	1.17	0.90–1.54
1–2 times/week	1.32^b^	1.07–1.62	1.30	1.01–1.67
1–2 times/month	1.15	0.90–1.47	1.07	0.79–1.45
Less than 1 time/month	1.64^a^	1.26–2.14	1.13	0.81–1.59
Never	3.53^a^	2.85–4.38	1.59^b^	1.20–2.10
High blood pressure				
No	1	–	1	–
Yes	1.21^b^	1.05–1.39	0.94	0.79–1.13
Stroke				
No	1	–	1	–
Yes	3.20^a^	2.20–4.63	3.41^a^	2.12–5.50

^a^p-value < 0.001

^b^p-value < 0.005

In the adjusted model, age, sex, educational attainment, physical exercise, main caregiver and stroke remained strongly associated with having cognitive symptoms of dementia. Meanwhile, the association between high blood pressure, mixed diet and cognitive symptoms became statistically non-significant. For the two factors of participating in social activities and visiting friends or neighbours, those who never engaged in such activities had higher odds of having cognitive symptoms of dementia by 2 times and 1.5 times respectively, compared to those who engaged in said activities daily.

## Discussion

This study was conducted to provide data on the general mental health status, as well as prevalence of dementia in the elderly in Vietnam. We hope that this study will bring attention for further research and investment in this field. With the cut-off point of 24, the prevalence of cognitive symptoms of dementia among the elderly was at 46.4% after standardisation according to age structure of the population census 2013. The prevalence found in this study was much higher than found by Tran Van Long’s study in 2013, which was 9.9% [6]. One explanation for this difference is that this study used a screening tool, MMSE, while Tran Van Long reported clinical diagnosis of dementia which are typically reserved for severe cases of dementia.

In this study, we found six factors that were strongly associated with cognitive symptoms of dementia including higher age, female biological sex, lower status of educational attainment, physical inactivity, lack of participation in social activities, and having had a history of stroke. Age is one of the most important risk factors of dementia. It has been found that risk of dementia escalates with increase in age. Only 1% of the population aged 60 to 64 suffered from dementia. After the age of 65, however, this proportion rose doubly for each 5-year period. The rate was at 1.4% among people aged 65 to 69; 2.8% to 4.1% in the 70 to 74 age group; 4.9% to 5.7% in the 75 to 79 age group; 8.7% to 13% among 80 to 84 age group and 16–25% in the group aged 85 or older [2, 3, 5]. This is also explained by biological mechanisms of aging that leads to reduced nerve function, and decline in motor functions. The progression of dementia would be faster and more severe as the body ages over time.

Association between educational attainment and dementia cognitive symptoms found in this study could be explained by the hypothesis of cognitive reserve. Matallana et al. [12] in an eleven years of follow-up analysis found that better educated people had higher MMSE score than those who had less years of education. Since cognitive impairment strongly associates with degradation of brain, the ability that brain can maintain the function could reduce risk of dementia. Higher education is an indication that the person has a larger cognitive reserve that could compensate the damage of the brain and maintain its function as usual. Additionally, people with lower educational level have fewer opportunities to access to learning resources and information such as nutritional care. Therefore, their ability to treat illness is limited, thus increasing the risk of cognitive impairment. Some authors, however, suggested that other factors including cultural environment and social context must be included to interpret the effects of such findings on tests [13, 14].

Being physically active and participating in social activities regularly helps the elderly maintain a healthy condition and prevent progression of such disease as dementia. Physical activities such as doing exercise or physical work helps the brain maintain an active state, thereby reducing the risk of function deficiency and dementia. In Vietnam, a study conducted by the Vietnam National Institute of Gerontology indicated that not having physical activity increased the risk of dementia by 2.3 times [15]. The absence of social activities limits the ability to communicate and impacts the memory of elderly. These activities include visiting friends or relatives, participating in clubs and religious activities. However, not participating in social activities can also be an early symptom and a predictor of cognitive impairment among the elderly. A previous study conducted in Vietnam found that lack of social activities and entertaining activities increased risk of dementia by 3.3 times and 1.7 times respectively [15].

Finally, as a direct cause of dementia, stroke damages the brain cells and directly affects functions like communication, movement and awareness, which in turn result in dementia. A study, which was conducted in 2009 among 285 patients with acute cerebral ischemic stroke at Cho Ray Hospital, showed that 42.4% of patients were at high risk of having dementia after a stroke. This relation was especially strong among group of people aged 60 or above [3].

## Strengths and limitations

This is one of a few studies conducted in Vietnam estimating prevalence of dementia and its cognitive symptoms among high-risk population. This population-based study sought to estimate the prevalence of cognitive symptoms of dementia, including undiagnosed cases. Participants were recruited from six communes which represents urban and rural area of three main regions of the country. Finally, in order to reduce bias, we conducted a pre-study to identify all eligible participants living in the study area.

Besides strengths, some weaknesses regarding methodology should be discussed. First, the relevance of the study results is limited to the study population due to the convenient sampling method. High refusal rate, 21.2%, possibly affects our findings as we have no information on the group who did not participate. However, the age distribution and sex ratio of our study sample was similar to the general elderly population in Vietnam according to the most recent census at the time of study [16]. The participant’s biological sex ratio was 148 females per 100 males while in the general population this ratio ranged between 113 and 200 females per 100 males [16]. In our sample, distribution of age groups was similar to the general Vietnamese elderly population (26.3%, 21.1%, 19.2%, 16.3% and 17.1% respectively to the age groups as in our study). Our sample had a slightly higher proportion of the age group of 80+ years old, which is known to have the highest risk of dementia. Hence, we calculated the age standardised prevalence using the general elderly population in Vietnam as standard population. Using the MMSE could overestimate the prevalence of dementia cognitive symptoms since the test result was suggested to vary across different level of education [13]. In term of associated factors, previous studies suggested that some other factors like being overweight and drug abuse [17] might increase risk of dementia but were not taken into account in this study.

## Conclusions

Proportion of older adults who have cognitive symptoms of dementia was high in Vietnam. While there are specific groups who are more vulnerable such as people with lower levels of education or those who have suffered from a stroke, some modifiable risk factors including physical inactivity and social engagement should also be considered in designing intervention for dementia prevention in the future. For the groups with high risk of dementia, promoting healthy lifestyle with increased physical and social activities could not only prevent dementia but also improve the quality of life of the elderly.

## Data Availability

The dataset generated during the current study are not publicly available due to protection of personal data within the study but are available from the corresponding author on reasonable request.

## Abbreviations

MMSE: Mini Mental State Evaluation
VND: Vietnam Dong
